# Imaging the Atomistic Dynamics of Single Proton Transfer and Combined Hydrogen/Proton Transfer in the O^–^ + CH_3_I Reaction

**DOI:** 10.1021/acs.jpca.2c06887

**Published:** 2022-12-13

**Authors:** Arnab Khan, Atilay Ayasli, Tim Michaelsen, Thomas Gstir, Milan Ončák, Roland Wester

**Affiliations:** Institut für Ionenphysik und Angewandte Physik, Universität Innsbruck, Technikerstrasse 25/3, 6020 Innsbruck, Austria

## Abstract

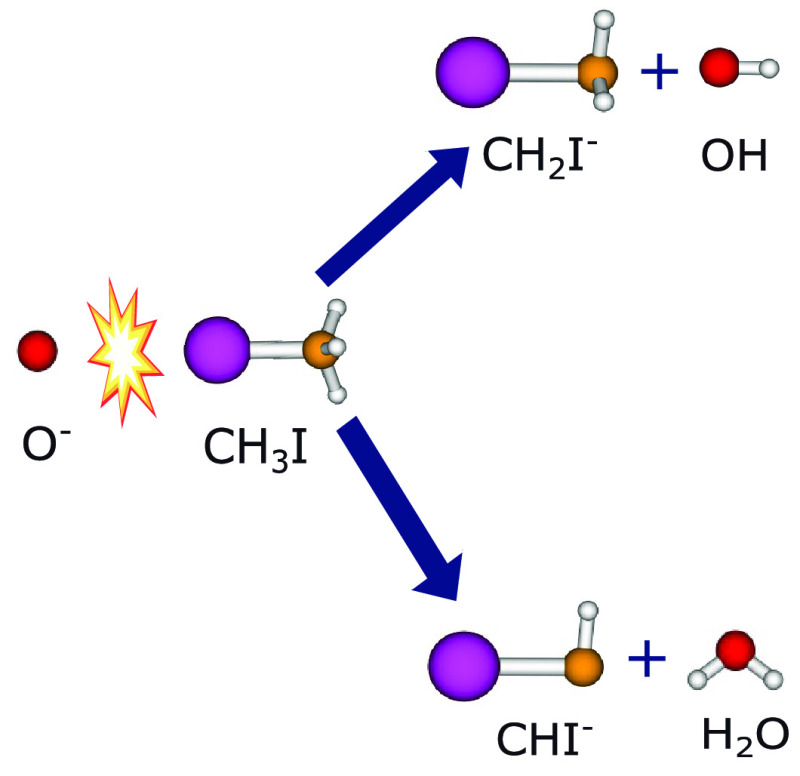

We report on reactive scattering studies of the proton transfer and combined hydrogen/proton transfer in the O^–^ + CH_3_I reaction. We combine state-of-the-art crossed-beam velocity map imaging and quantum chemistry calculations to understand the dynamics for the formations of the CH_2_I^–^ + OH and CHI^–^ + H_2_O products. The experimental velocity- and angle-differential cross section show for both products and at all collision energies (between 0.3 and 2.0 eV) that the product ions are predominantly forward scattered. For the CHI^–^ + H_2_O channel, the data show lower product velocities, indicative of higher internal excitation, than in the case of single proton transfer. Furthermore, our results suggest that the combined hydrogen/proton transfer proceeds via a two-step process: In the first step, O^–^ abstracts one H atom to form OH^–^, and then the transient OH^–^ removes an additional proton from CH_2_I to form the energetically stable H_2_O coproduct.

## Introduction

Gas-phase studies of neutral–neutral^1−5^ and ion–neutral^6−11^ reactive scattering at the level of single atoms allows one to gain insights into the atomistic dynamics of many classes of chemical reactions. An overwhelming growth has been observed in the field of gas-phase reaction dynamics research over the past decades. The crossed molecular beam technique, in particular when equipped with velocity map imaging (VMI),^12^ has proven its potential to explore the dynamics of gas-phase reactions under single-collision conditions.^13−17^ The VMI spectrometer enables accurate velocity measurements of the reactants and products with a full 4π solid angle collection efficiency. In this way, one can obtain kinematically complete information about elementary reaction processes.

It has been observed that in X^–^ + R–Y type anion–molecule reactive scattering, a manifold of reaction pathways is possible. Besides the well-known S_N_2 channel, other competing reaction channels open up with suitable experimental conditions. Generally, this depends on the ion species, collision energy, angle of attack, and the short-range interaction. For example, in the in F^–^ + CH_3_I or OH^–^ + CH_3_I systems, proton transfer (CH_2_I^–^ formation) and halide abstractions (FI^–^, FHI^–^, and IOH^–^ formation) have been observed experimentally.^18−20^ These channels have also been studied for the Cl^–^ + CH_3_F system.^21^ The experimental results are in good agreement with calculations.^4,22,23^ Specifically the proton transfer reaction is of interest as it occurs in different biological and chemical processes and can be recognized as one of the essential reactions in nature.^24−32^

When instead of a closed shell anion, reactions of the radical anion O^–^ with different organic molecules, such as halogenated alkanes, are probed, an additional channel opens up due to the ability of O^–^ to abstract two protons and an electron from the molecules.^33−35^ As a result, it forms the thermodynamically very stable water product. Here we refer to this process as “combined hydrogen/proton” transfer. Such a combined hydrogen/proton abstraction reaction is not energetically allowed for halide anions reacting with organic molecules. In reactions of O^–^ with small alkanes at room temperature the reaction proceeds mainly via H atom abstraction forming OH^–^ product ions.^36^ Crossed-beam studies have been performed to study the product energy disposal and angular distributions for O^–^ reacting with methane.^10^ For O^–^ reacting with acetylene single proton transfer and the formation of a C–O bond were the dominant product channels.^37^ The reaction dynamics of proton and hydrogen/proton transfer of O^–^ from halogenated hydrocarbons have not been studied to date. Such a study is interesting for the understanding of O^–^ radical-induced C–H bond activation in larger hydrocarbons.^38^

Here we study proton and combined hydrogen/proton transfer in the O^–^ + CH_3_I reaction. This allows us to probe the collision-energy-dependent evolution of the proton and combined hydrogen/proton transfer reactions and to investigate possible correlations between these channels. We aim to understand the dynamical aspects of the two transfer reaction pathways, which lead to the formation of CH_2_I^–^ and CHI^–^ product ions. Until now, most of the O^–^ + CH_3_Y systems have been studied using the flow-tube technique.^34^ However, using the flow-tube technique, one cannot probe highly endoergic reactions as these measurements are done at or close to room temperature conditions. Therefore, no proton transfer has been seen in the flow-tube measurements on O^–^ + CH_3_Y.^34^

## Experimental Methods

The presented measurements have been performed with an ion–molecule crossed-beam setup equipped with a VMI spectrometer. Readers can find a detailed discussion on the experimental technique elsewhere.^39,40^ Here, we will describe the information relevant to this present study in a gist. The O^–^ ions are produced in a pulsed plasma discharge of about 1–2% N_2_O in argon. These ions are accelerated in a Wiley–McLaren type mass spectrometer and guided into an octupole ion trap, where they thermalized to room temperature. Then the ions are extracted from the trap and crossed with the helium-seeded CH_3_I beam at an angle of about 60°. These two beams cross each other at the center of the VMI spectrometer. Note that after the extraction the ions are decelerated to the desired experimental kinetic energy by several electrostatic lens combinations. We have chosen eight relative collision energies between 0.3 and 2.0 eV in the center-of-mass frame for this study. The entire experiment operates at a 20 Hz repetition rate.

Following the ion–neutral scattering, we image the position and time-of-flight of the product ions with the VMI spectrometer. The position is converted in to the translational product velocity parallel to the detector plane. From the time-of-flight we determine the mass of the particular product anion and, after integration over many events, the product branching ratios. From the difference of the time-of-flight to its average value for the respective anion mass, we compute its vertical velocity component. From the three-dimensional velocity vectors for all measured product ions, we produce two-dimensional scattering images in a plane containing the relative velocity axis as well as one-dimensional angular distributions.

## Results

**Figure 1:**
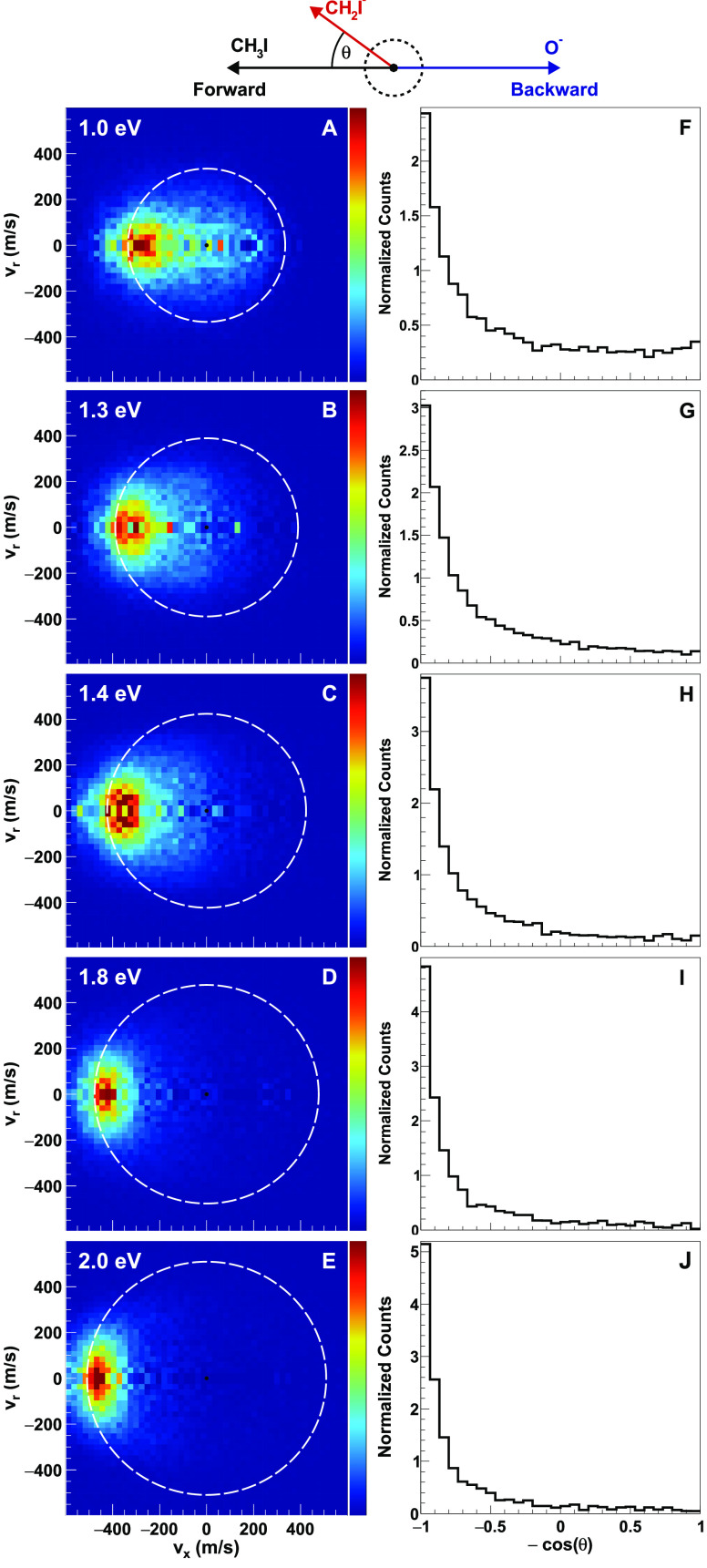
(A–E) O^–^ + CH_3_I → CH_2_I^–^ + OH channel (proton transfer) product center-of-mass frame velocity distributions of the CH_2_I^–^ product at different collision energies. At the top of the images, the Newton diagram illustrates the relative orientation of the velocity vectors of the reactants and the charged product ions. The white dotted circle in each velocity image gives the kinematic cutoff for proton transfer reaction. (F–J) Velocity integrated angular distributions for CH_2_I^–^ ions.

Here we present results for the two proton transfer product ions CH_2_I^–^ and CHI^–^ from the radical anion–molecule reaction O^–^ + CH_3_I. In Figures 1A–E, we show first the reactive scattering cross section for the proton transfer product CH_2_I^–^ at different collision energies. As explained above, the images represent a slice through the full three-dimensional velocity distribution in the center-of-mass frame, representing the relative differential cross section in the scattering plane. The proton transfer channel starts to appear around a collision energy *E*_col_ of 0.7 eV, but we show images only from 1.0 eV onward as at lower collision energy not enough statistics could be acquired. The velocity distributions reveal a strong forward scattering feature for all collision energies; i.e., the scattering events concentrate at the left side of the images. At low collision energies, isotropic scattering with low product velocity can be seen, which is expected to imply a long-lived complex-mediated mechanism. The same feature is also evident in the angular distribution plots shown in Figures 1F–J. In addition, both the velocity and angular distribution plots show that as *E*_col_ increases, the indirect contribution decreases, and the distributions become fully forward-dominated. The white dashed circles drawn in the velocity images show the kinematic cutoff values for each collision energy, which is the maximum product velocity considering energy and momentum conservation and including the calculated reaction endoergicity (see Discussion section and Figure 4). The observed signal outside the kinematic cutoff is a result of the finite experimental energy resolution.

**Figure 2:**
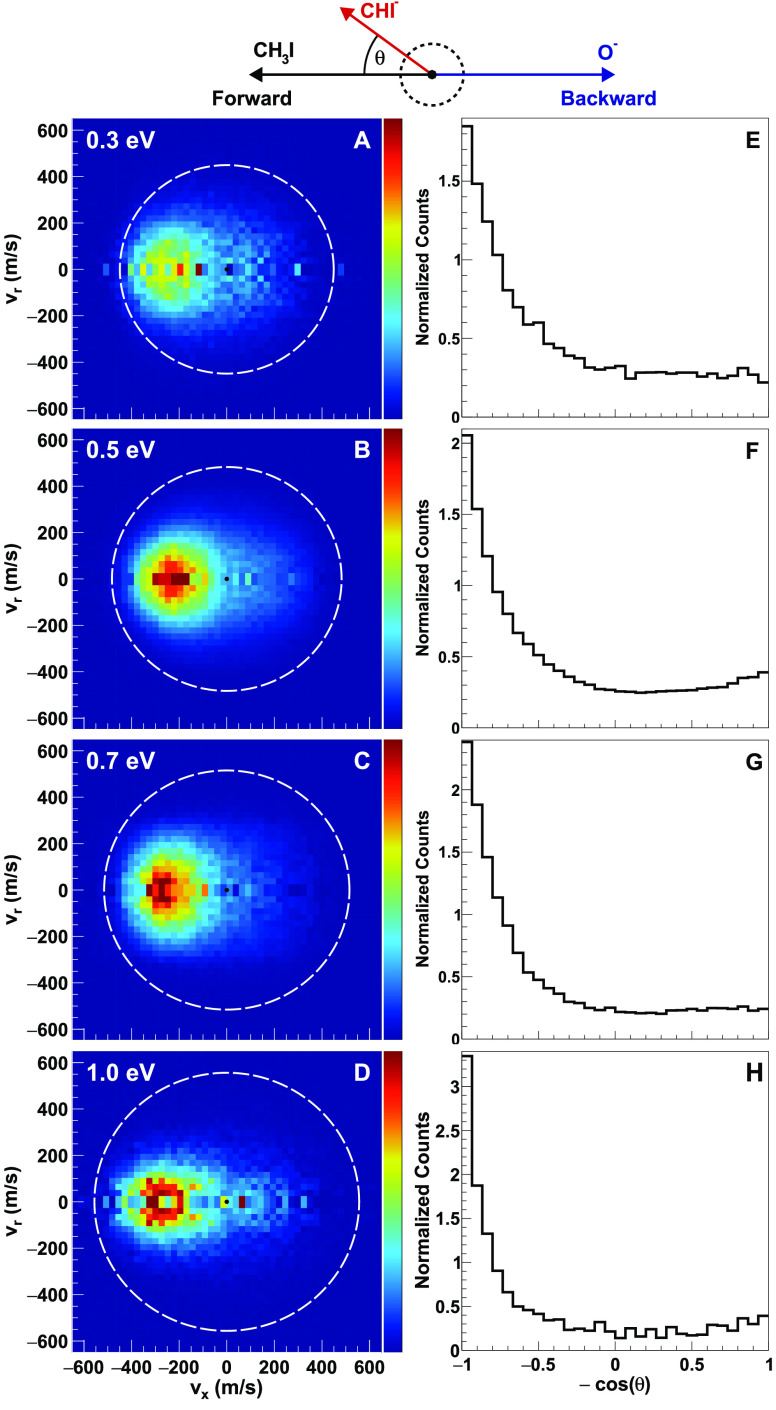
(A–D) Center-of-mass frame velocity distributions of the CHI^–^ ions from the O^–^ + CH_3_I → CHI^–^ + H_2_O (combined hydrogen/proton transfer) channel at different relative collision energies. The Newton diagram shown at the top of the experimental data represents the relative orientations of all the velocity vectors of the reactants and the CHI^–^ product ions. The white dotted circle shows the kinematic cutoff values for the combined hydrogen/proton transfer pathway. (E–H) Velocity integrated angular distributions for CHI^–^ products.

The velocity and angular distributions for the product anion CHI^–^ are plotted in Figures 2A–D. These ions are formed after the transfer of two protons and one electron, termed “combined hydrogen/proton transfer” above. Here we show the data for four experimental collision energies between 0.3 and 1.0 eV. For collision energies above 1.0 eV the event counts for this channel drop rapidly and become statistically insignificant. Therefore, we cannot show scattering images for collision energies of 1.3 eV and higher. We can see that the velocity distributions show distinct forward scattered features at all plotted energies. In the velocity distributions, we also see some isotopically scattered counts at the center of the images, which correspond to an indirect mechanism. However, at all studied collision energies, the forward scattering events dominate over the indirect pathways. Interestingly, the velocity magnitudes of the forward scattered products are significantly smaller than the kinematic cutoff, which is shown in the images by the white circle. This is markedly different for the CH_2_I^–^ products, which are scattered into product velocities close to the kinematic cutoff. The CHI^–^ forward velocities also show a larger spread in comparison with the CH_2_I^–^ product velocities. In the right panels, Figures 2E–H, we show the integral angular distributions of the product ion. The angular distributions also reflect the strong forward scattering behavior of the CHI^–^ products along with the isotropic indirect pathway.

**Figure 3:**
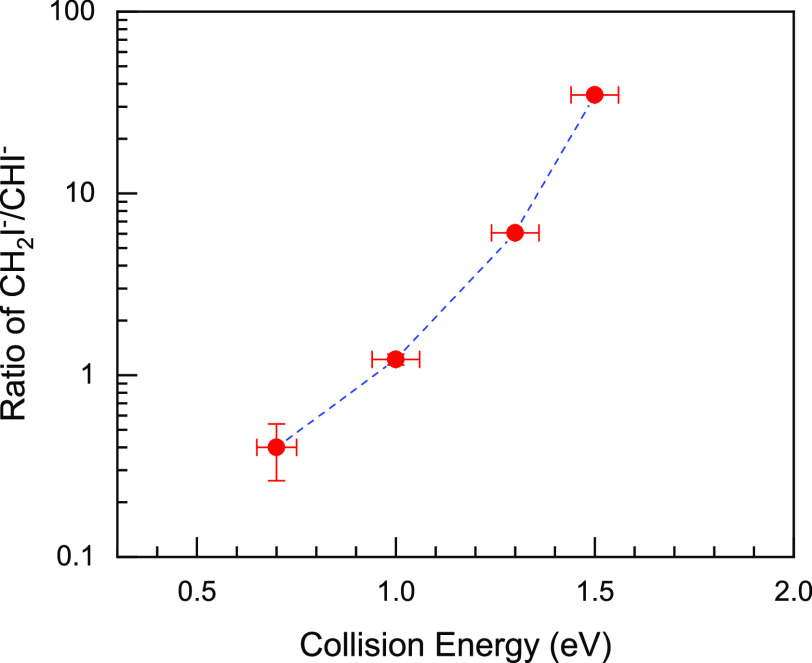
Collision-energy-dependent relative branching ratio of the CH_2_I^–^ and CHI^–^ ions. The relative branching ratio is obtained by dividing the branching ratio of the CH_2_I^–^ and CHI^–^ ions. The error on the collision energy stems from the uncertainty in measuring the velocity vectors of the O^–^ and CH_3_I reactant beams. The error on the branching ratio represents the statistical accuracy.

Additional information about the studied reaction can be obtained from the relative branching ratios for the different reaction products as a function of the collision energy. Here we focus on the two products CH_2_I^–^ and CHI^–^, which together carry between 10% and 25% of the total measured flux. For the reaction of O^–^ ions with CH_3_I we have also observed the product ions I^–^ and IO^–^, which amount to between 75% and 90% of the flux. Their dynamics are presented in a separate publication.^41^ In the present experiment we could not detect OH^–^ product ions due to a contamination from ^17^O and/or OH^–^ in the reactant ion beam that could not be sufficiently mass gated. The relative branching ratio of CH_2_I^–^ and CHI^–^ is shown in Figure 3. As the ratio changes strongly with collision energy, we plot it on a logarithmic scale. For *E*_col_ lower than 0.7 eV the proton transfer channel is energetically closed and the logarithmic value is undefined. The same is true for *E*_col_ = 1.8 eV or higher, where the CHI^–^ channel becomes unobservable. Therefore, Figure 3 shows the branching ratio in the range from 0.7 to 1.5 eV. We notice that as the collision energy increases, the relative branching of single proton transfer to combined hydrogen/proton transfer increases rapidly.

## Discussion

The images of the differential reaction cross section show that both CH_2_I^–^ and CHI^–^ are scattered in the forward direction with little momentum transfer. This implies that the collisions occurred predominantly at large impact parameters. Single proton transfer led to very small energy transfer to internal degrees of freedom of the CH_2_I^–^ rovibrational degrees of freedom, which is clear from the large product velocities close to the kinematic cutoff. Combined hydrogen/proton transfer, on the other hand, shows lower product velocities indicative of stronger rovibrational coupling in the reaction complex that transfers some of the available energy into the internal states of either CHI^–^ or H_2_O or both. The combined transfer is favored at low collision energies and becomes strongly suppressed at a collision energy of 1.5 eV while single proton transfer takes over.

**Figure 4:**
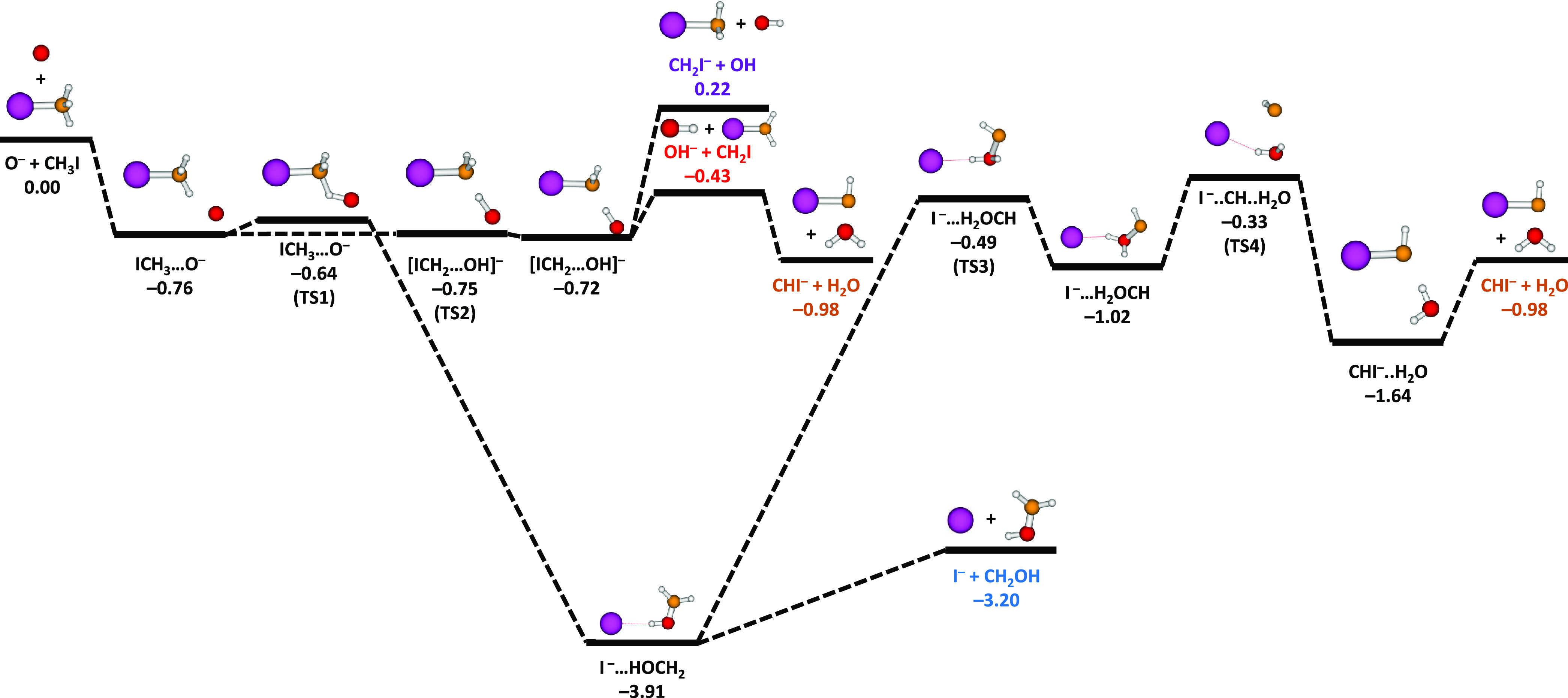
Energy levels and minimum-energy pathways of different reaction channels calculated at the CCSD(T)/aug-cc-pVTZ(-PP)//MP2/aug-cc-pVTZ(-PP) level. The abbreviation “TS” represents the transition state. All energies are given in the units of eV.

To yield a better insight into the experimental observations, we have performed *ab initio* quantum mechanical calculations using the CCSD(T)/MP2 level of theory. Wave function stability with respect to relaxing various constraints was tested prior to every calculation, and if an instability appeared, the wave function was stabilized, using a procedure implemented in the Gaussian package. After that all isomers were optimized at the MP2 level using the aug-cc-pVTZ-PP basis set for the I atom and aug-cc-pVTZ for the other atoms with a subsequent single-point energy recalculation at the CCSD(T) level in the optimized structure. All relative energies are zero-point energy corrected using vibrational frequencies calculated at the MP2 level. We performed the calculations using the Gaussian 16 package.^42^ The energetics and detailed reaction pathways for the formation of CHI^–^ and CH_2_I^–^ are shown in Figure 4. In addition, Figure 4 shows the pathways that lead to I^–^ + CH_2_OH and OH^–^ + CH_2_I products. Both pathways are exoergic with an exoergicity of −3.20 and −0.43 eV, respectively. Note that from the OH^–^ + CH_2_I product channel a barrier-free pathway exists to form H_2_O + CHI^–^. Unfortunately, as stated above, we could not detect OH^–^ product ions in the present experiment.

The *ab initio* calculations show that as O^–^ approaches CH_3_I, it gets at first attracted into the ICH_3_...O^–^ well. From there, the single proton transfer takes place by crossing TS2, which is an essentially barrierless pathway given that the computed value for the barrier height of 0.01 eV is smaller than the expected accuracy of the *ab initio* calculations. The products CH_2_I^–^ + OH are formed with a computed exoergicity of 0.22 eV, which is in qualitative agreement with the experimental opening of this channel above 0.7 eV collision energy. At low collision energies, the isotropic part of the CH_2_I^–^ product velocity distribution (in particular in Figures 1A,F) suggests complex formation with a lifetime comparable to its rotational period. With the increase in collision energy, as the interaction time becomes smaller, the CH_2_I^–^ distribution shifts toward mostly forward-directed scattering.

Very similar tendencies have been observed for the proton transfer reaction of OH^–^ + CH_3_I^19^ and F^–^ + CH_3_I^18^ systems. Like in these two systems, a hydrogen-bonded prereaction complex forms in the present case. However, the O^–^ + CH_3_I system is even more similar to F^–^ + CH_3_I as in both cases the proton transfer reaction is endoergic. On the other hand, the proton transfer reaction OH^–^ + CH_3_I → CH_2_I^–^ + H_2_O is slightly exoergic in character as it leads to the stable water coproduct. This case is similar to the exoergic O^–^ + CH_3_I → CHI^–^ + H_2_O channel in this study. Furthermore, for the O^–^ + CH_3_I → CH_2_I^–^ + OH channel the peak of the velocity distributions lies very close to the calculated kinematic cutoff value, suggesting a faster reaction and fairly inefficient energy coupling between different rovibrational modes during single proton transfer. It has been seen before for the proton transfer reactions of F^–^ and OH^–^ with CH_3_I that the neutral coproduct generally remains in the vibrationally ground state, while a small part of the available energy is deposited into the vibrational and rotational modes of the CH_2_I^–^ ion.^18,19,22^

Two pathways lead to the formation of CHI^–^ by combined hydrogen/proton transfer, a channel that is exoergic with Δ*E* = −0.98 eV. From the initial ICH_3_...O^–^ well the first reaction pathway leads across the submerged transition state TS1 to the post-reaction complex after oxygen insertion into the CH_3_I skeleton. Instead of forming I^–^ + CH_2_OH products, this reaction complex may rearrange by an H migration giving rise to the TS3 of the shape [I···H_2_OCH]^−^. At the next transition state TS4, a new C–I bond forms and finally leads to the CHI^–^ + H_2_O channel. The other pathway to CHI^–^ proceeds across the shallow TS2 by formation of OH^–^ + CH_2_I as intermediate products. Instead of separating, the OH^–^ product may abstract a proton from the CH_2_I radical. For this last step we have not found an intermediate barrier.

The first pathway to CHI^–^ progresses via several bond formation and cleavage steps and also a higher energy transition state (TS4), which makes it less probable compared to the more direct pathway via OH^–^ formation. Furthermore, a relatively more efficient vibrational and rotational energy redistribution can be expected for this pathway, which is in contrast to the predominantly direct forward scattering observed in the measurement. We therefore assume that at low collision energies the combined hydrogen/proton transfer proceeds preferentially via H atom transfer to form OH^–^, followed by a second proton transfer step. It has been noted before that the H atom affinity of O^–^, which leads to the formation of OH^–^ in reactions with alkanes, may be an intermediate step in the formation of H_2_O as the final product.^33^ Our analysis supports this prediction. For the second proton transfer a sufficient amount of interaction time of OH^–^ with CH_2_I is required. This and the additional bending vibrational degree of freedom in the H_2_O product make it more likely that vibrational energy is stored in internal degrees of freedom of the two product molecules. This can explain the lower measured product velocities (see Figure 2 compared to Figure 1).

The measured energy-dependent relative branching ratio for the CH_2_I^–^ versus CHI^–^ channels supports the above discussion. When *E*_col_ is low, the transfer reaction fully favors the exoergic combined hydrogen/proton transfer pathway. However, as the collision energy increases, the OH^–^ product has a higher chance of leaving the OH^–^ + CH_2_I system before the pathway to CHI^–^ + H_2_O is completed. The single proton transfer then rapidly dominates over the combined hydrogen/proton transfer.

## Conclusions

We report on the dynamics of single proton transfer and combined hydrogen/proton transfer reactions for radical anion O^–^ with CH_3_I molecules using a crossed-beam velocity map imaging setup. The data have been analyzed with the aid of *ab initio* calculations. The velocity and angle differential reaction cross sections have been obtained as a function of collision energy. These scattering images show a strong preference for direct forward scattering in both cases. When the collision energy is low an isotropic indirect scattering distribution can also be seen, which eventually decreases with the increase in collision energy. The indirect mechanism is somewhat stronger in the case of the combined hydrogen/proton transfer reaction, which points at a more efficient coupling between vibrational and rotational modes in this case compared to the proton transfer reaction. With increasing collision energy the relative branching increases strongly for the single proton transfer. On the basis of the presented measurements and calculations, we explain the combined hydrogen/proton transfer as a two-step process: hydrogen abstraction by the incoming O^–^ followed by proton transfer of the transient OH^–^ product.

## Supporting Information Available

Cartesian coordinates and zero-point-corrected energies of calculated molecular structures (PDF)
