# Supplementary material for: Imaging the Atomistic Dynamics of Single Proton Transfer and Combined Hydrogen/Proton Transfer in the O– + CH3I Reaction
Source: J Phys Chem A. 2022 Dec 13;126(50):9408–13. doi: 10.1021/acs.jpca.2c06887 (PMC9791656; doi:10.1021/acs.jpca.2c06887)
Supplement: Supplementary file 1 — jp2c06887_si_001.pdf [file jp2c06887_si_001.pdf]

Supporting Information

for

Imaging the atomistic dynamics of of single proton transfer and combined hydrogen/proton transfer in the O^{-} + CH_3I reaction

Arnab Khan, Atilay Ayasli, Tim Michaelsen, Thomas Gstir,
Milan887Ončák, and Roland Wester∗

Institut für Ionenphysik und Angewandte Physik, Universität Innsbruck,
Technikerstraße 25/3, 6020 Innsbruck, Austria

Electronic mail: roland.wester@uibk.ac.at

Cartesian coordinates (in Å) and zero-point corrected energies (in Hartree) of various ions and molecules optimized at the MP2/aug-cc-pVTZ(-PP) level

```
O⁻
E = -75.011329
o 0.000000 0.000000 0.000000

CH₃I
E = -334.659616
I -0.000001 0.324146 0.000000
C -0.000001 -1.799162 0.000000
H -1.031804 -2.128261 0.000000
H 0.515943 -2.128254 0.893559
H 0.515943 -2.128254 -0.893559

ICH₃…O⁻
E = -409.698207
i -0.729693 -0.100642 -0.000000
c 1.107032 0.999160 -0.000002
o 3.489156 -0.520202 0.000001
h 1.976236 0.267767 -0.000221
h 1.070943 1.616616 -0.892867
h 1.071132 1.616279 0.893098

ICH₃…O⁻ (TS1)
E = -409.688720
o 3.254494 -0.365650 0.000000
c 1.322970 0.620476 0.000000
h 2.125092 -0.428479 0.000000
h 1.425142 1.211569 0.900843
h 1.425142 1.211568 -0.900843
i -0.734890 -0.052685 0.000000

I-…HOCH₂
E = -409.820956
o 2.448216 -0.698201 0.008532
h 4.024227 0.603458 0.192503
c 2.976876 0.544141 -0.059387
h 2.312384 1.384452 0.080125
h 1.462017 -0.600230 -0.022945
i -0.853691 0.017605 0.000724

[ICH₂…OH]⁻ (TS2)
E = -409.698607
c -0.858081 -1.279053 0.000000
o 0.996536 -3.141990 0.000000
h 0.186501 -2.253878 0.000000
h -1.505151 -1.258662 0.882266
h -1.505151 -1.258662 -0.882266
i -0.000000 0.709084 0.000000

[ICH₂…OH]⁻
E = -409.696823
C -0.918743 -1.264878 0.000000
I 0.000000 0.712869 0.000000
H -1.575400 -1.193526 0.876591
H 0.303572 -2.406034 0.000000
H -1.575400 -1.193526 -0.876591
O 1.044961 -3.174963 0.000000

CH₂I-
E = -334.043328
I 0.021648 -0.295592 0.000000
```

C 0.021648 1.914142 -0.000000
H -0.638629 2.090756 0.867700
H -0.638629 2.090756 -0.867700

OH
E = -75.617695
O 0.000000 0.000000 0.107729
H 0.000000 0.000000 -0.861836

OH^-
E = -75.693651
O 0.000000 0.000000 0.107513
H 0.000000 0.000000 -0.860108

CH_2I
E = -334.001430
I 0.002159 -0.282789 -0.000000
C 0.002159 1.745596 0.000000
H -0.063703 2.257131 0.942794
H -0.063703 2.257131 -0.942794

I^-
E = -294.965831
I 0.000000 0.000000 0.000000

CH_2OH
E = -114.828514
o -0.670344 -0.125770 0.021786
h 1.228740 -0.883135 0.109462
c 0.684839 0.027454 -0.066718
h 1.113350 0.987789 0.174110
h -1.088370 0.736781 -0.057552

I–…H_2OCH (TS3)
E = -409.693391
o 2.354960 -0.722285 -0.099035
h 2.886805 -0.368823 0.808981
c 3.134164 0.692569 0.020610
h 2.348654 1.396669 -0.276159
h 1.360751 -0.516980 -0.043362
i -0.834733 0.020981 0.003380

I–…H_2OCH
E = -409.711149
o 2.310097 -0.553485 -0.158414
h 2.373453 -1.194213 0.558821
c 3.453059 0.510751 0.160057
h 3.010666 1.341822 -0.420559
h 1.339342 -0.228964 -0.137134
i -0.866464 0.027259 0.005771

I^-…CH…H_2O (TS4)
E = -409.676390
c -2.549879 2.057123 -0.015608
o -2.400404 -1.453037 0.087679
i 0.760967 0.013111 -0.002029
h -1.890849 1.185156 0.151683
h -1.484098 -1.128619 0.220580
h -2.453776 -1.469848 -0.872506

I^-…CH…H_2O
E = -409.734945
C 1.136130 -1.149272 0.000000
I 0.000000 0.709223 0.000000
H 2.159726 -0.730519 0.000000
O -0.906914 -3.121233 0.000000
H -0.101077 -2.516861 0.000000
H -1.620118 -2.475917 0.000000

CHI^-
E = -333.401607
I 0.018462 -0.259968 0.000000
C 0.018462 1.950442 0.000000
H -1.089230 2.075648 0.000000

H_2O
E = -76.307582
O 0.000000 0.000000 0.118231
H -0.000000 0.758132 -0.472922
H -0.000000 -0.758132 -0.472922
